# Maternal Obesity: A Focus on Maternal Interventions to Improve Health of Offspring

**DOI:** 10.3389/fcvm.2021.696812

**Published:** 2021-07-21

**Authors:** Akriti Shrestha, Madison Prowak, Victoria-Marie Berlandi-Short, Jessica Garay, Latha Ramalingam

**Affiliations:** Department of Nutrition and Food Studies, Syracuse University, Syracuse, NY, United States

**Keywords:** maternal obesity, nutritional interventions, physical activity, high fat diet, animal models

## Abstract

Maternal obesity has many implications for offspring health that persist throughout their lifespan that include obesity and cardiovascular complications. Several different factors contribute to obesity and they encompass interplay between genetics and environment. In the prenatal period, untreated obesity establishes a foundation for a myriad of symptoms and negative delivery experiences, including gestational hypertensive disorders, gestational diabetes, macrosomia, and labor complications. However, data across human and animal studies show promise that nutritional interventions and physical activity may rescue much of the adverse effects of obesity on offspring metabolic health. Further, these maternal interventions improve the health of the offspring by reducing weight gain, cardiovascular disorders, and improving glucose tolerance. Mechanisms from animal studies have also been proposed to elucidate the signaling pathways that regulate inflammation, lipid metabolism, and oxidative capacity of the tissue, ultimately providing potential specific courses of treatment. This review aims to pinpoint the risks of maternal obesity and provide plausible intervention strategies. We delve into recent research involving both animal and human studies with maternal interventions. With the increasing concerning of obesity rates witnessed in the United States, it is imperative to acknowledge the long-term effects posed on future generations and specifically modify maternal nutrition and care to mitigate these adverse outcomes.

## Introduction

Obesity is increasing at alarming rates with a current prevalence of more than 42% in US adults and 17% in children (1). Further, around 50% of US women of reproductive age (20–39 years) are either overweight (25.6%) or suffer from obesity (24.8%) (2). Maternal obesity is recorded when the prepregnancy BMI exceeds 30. Significant differences in maternal obesity prevalence exist among different races and ethnicities in the USA (1). Higher rates of maternal obesity were observed among Black and American Indian/Alaskan Native women with 33.9 and 34.9% suffering from it, respectively (3). Comparatively, 27% of Caucasian women and 27.4% of Asian American women have maternal obesity. These data suggest that maternal obesity is certainly a cause for concern deserving further research. Interventions during this critical period could promote better health outcomes in the offspring. In this review, we have reviewed the impact of maternal obesity, followed by nutritional interventional studies in humans. The discussion on human studies is followed by that on animal studies undertaken to gain mechanistic insights on maternal programming effects in the offspring. Furthermore, we present findings in regard to the role of physical activity and its impact on reducing adverse pregnancy complications. Overall, a number of studies performed in animal models are discussed to draw a comprehensive conclusion on the role of maternal intervention on the improvement of offspring health.

Multiple environmental factors contribute to obesity such as diet, physical activity, and mental stress (4). In addition to environmental influences, epigenetic programming could intensify severity of obesity, inducing several health issues in the offspring (5–10). Various non-modifiable factors like pregestational body weight, maternal height and age, number of births, socioeconomic status, and ethnicity are associated with maternal obesity (4).

Apart from obesity status (as determined by BMI), another critical parameter of maternal health status is physical activity level since it has the potential to influence gestational weight gain (GWG). Thus, engaging in exercise during pregnancy is especially important for women with a previously sedentary lifestyle since inactivity by itself can lead to impaired cardiovascular function and obesity as discussed in subsequent sections (11, 12). A third factor is preexisting disease condition in the mother such as hypothyroidism, polycystic ovary syndrome, and/or Cushing's syndrome many of which are related to maternal obesity (13–15). Furthermore, medications such as antidepressants, diabetes treatments (insulin), oral contraceptives, and hypertension medicines are also associated with maternal obesity; the strength of the associations, varies depending on individual response to the medication (16). The impacts of preexisting disease and medications on maternal obesity are reviewed elsewhere (13–15).

## Complications of Maternal Obesity in the Mother

Excessive GWG is associated with labor complications, cesarean section rates, postpartum maternal weight retention, preeclampsia, macrosomia, and increased BMI in offspring throughout their life (11, 17). In line with this, a prospective study completed in 2018 compared nulliparous women having metabolic syndrome (MS) with their healthy counterparts and found that over half of the MS group experienced at least one complication in their pregnancy, whereas only 33% of women in the healthy group experienced one complication (18). The complications included pulmonary embolism, gestational diabetes mellitus (GDM), or hypertension. Other complications arising from excessive GWG can involve preterm delivery, induction of labor, instrumental vaginal delivery, longer labor and delivery times, stillbirths, postnatal fetal demise within 7 days, and NICU admissions (19, 20). Further, a 2018 meta-analysis indicated that risk of shoulder dystocia—a potentially fatal complication during labor—is significantly increased with maternal obesity (20, 21). In the preconception stage, maternal obesity decreases fertility as reflected in the increased length of time required to conceive. Further, there is increased incidence of miscarriage and significantly higher risk for postpartum hemorrhage among obese mothers (20).

Maternal obesity increases risk of GDM, which is exacerbated by decreased or complete lack of physical activity (22–24). A 2015 review showed that 15–45% of babies born to mothers with GDM have macrosomia, compared to 12% for healthy-weight mothers (25). This is likely due to impaired glucose tolerance observed in GDM; the increase in maternal blood glucose levels causes an increase in fetal blood glucose levels as the excess glucose passes across the placenta and enters fetal circulation. Not all of this glucose is used for fetal metabolism, leading to increased storage which ultimately contributes to macrosomia (25). Further, babies born to mothers with untreated GDM are at increased risk of impaired insulin resistance (IR) and decreased beta-cell functionality throughout their lives (26). Though the true prevalence of GDM in the United States is unknown, NHANES data from 8,185 participants between 2007 and 2014 showed that 7.6% of women had GDM. Also, 19.7% of women who had GDM were ultimately diagnosed with type 2 diabetes (T2D) postpartum (27). Increasing physical activity in women of childbearing age is currently recognized as one strategy to reduce GDM and its comorbidities (28).

### Maternal Obesity and Cardiovascular Health

Plenty of available evidence points toward significant associations between maternal obesity and offspring cardiometabolic health (4, 29–32). These studies are based on the “developmental overnutrition hypothesis” that explains the association between mother and child's obesity. According to Armitage et al. (29), higher circulating levels of glucose, free fatty acids (FFA), amino acids, (29) fetal insulin (16), and digestive hormones (33) in the mother cause a permanent alteration of appetite control and energy metabolism in the fetus. Further, this is transferred to the next generation through inheritance. Additionally, this leads to hypertension and vascular dysfunction along with dysregulation of glucose/insulin homeostasis in offspring (30). The modifications observed in the offspring of obese mothers including alteration in muscle structure, appetite, and physical activity support the programming hypothesis of maternal obesity (30). However, the detailed mechanism(s) involved in such inheritance is not completely understood.

Maternal obesity is also strongly associated with elevated risk of cardiovascular anomalies, hydrocephaly, neural tube defects as well as cardiac dysfunction during the fetal development stage (10). Moreover, hyperglycemia during pregnancy is a major risk factor that alters fetal myocardium and cardiovascular programming in the offspring which affects adult life. There are several mechanisms by which hyperglycemia alters the cardiovascular programming. One of the mechanisms is via the production of reactive oxidative species (ROS) (34–36), which stimulates inflammatory signal transduction cascades leading to impaired cardiovascular programming. Other possible mechanisms include lipid peroxidation, endoplasmic reticulum stress, and hypoxia (37, 38). Reactive oxidative species also causes excess lipid peroxidation and cardiac fibrosis which is another reason behind dysfunctional cardiovascular programming (35, 39, 40). Bayoumy et al. (41) measured cardiac function in fetuses of obese, diabetic, and normal pregnant women by employing tissue Doppler imaging at 30 weeks gestation. Fetuses of obese and diabetic pregnant women had lower systolic and diastolic function compared to women with normal BMI, indicating weakened cardiovascular function in developing fetuses owing to maternal obesity. These observations of cardiovascular effects on offspring are difficult to attribute in entirety to abnormal glucose metabolism in obese mothers. Thus, further investigation is warranted to better understand the overall impact of maternal obesity on fetal health status (41).

These observations prompted a study by Persson et al. (42) which investigated the association of mother's obesity with specific heart defects in the child. They examined congenital defects like tetralogy of Fallot (a special type of congenital heart disease made up of four defects: ventricular septal defect, pulmonary stenosis, overriding aorta, and ventricular hypertrophy) (43), transposition of the great arteries, aortic arch defects, atrial septal defects (ASD) as well as single-ventricle heart in the children to determine if there was an association of any of these conditions with maternal BMI. It was observed that the adjusted parameter of prevalence rate ratios (PPRs) of aortic branch defect, persistent ductus arteriosus and ASD concomitantly increase with the rise in severity of maternal obesity, establishing a direct relationship between specific heart defects in children with maternal obesity for the very first time.

Studies have indicated that excessive GWG leads to not only maternal obesity but also higher blood pressure and inflammatory markers in children (5–9). In particular, excessive GWG was directly associated with adverse effects on offspring cardiovascular health (10). Offspring born to mothers suffering from obesity had higher odds ratios (OR) of cardiovascular anomalies (OR: 1.30), neural tube defects (OR: 1.87), and septal anomalies (OR: 1.20) (10). Another study performed in Greece with 977 mother–child pairs showed that excessive GWG in the first trimester of pregnancy correlated with higher diastolic blood pressure in children at 4 years of age (44). Corroborating this, a study conducted among 2,432 mother–child dyads in Australia indicated similar strong associations between systolic blood pressure in the offspring at 21 years of age with excessive GWG in the mother (45). Further, an elegant study (46) with 37,709 participants revealed that maternal obesity is associated with significantly greater risk of hospital admissions for cardiovascular-related issues during adulthood of offspring born to obese mothers. Collectively, these results imply that maternal obesity and excessive GWG have a significant impact on the future cardiovascular health of the offspring. The exact mechanism(s) that govern this influence is/are not well-understood, and could be attributed to epigenetic modifications or other factors like environmental factors during early stage of life (47). It is difficult to shed light on direct effects of maternal obesity on offspring body composition and cardiometabolic health because of postnatal lifestyle choices and environmental influences on the offspring, which are harder to distinguish from genetic effects. In line with this, additional factors like duration and time of exposure to an obesogenic maternal diet should be considered because critical time points during which maternal obesity influences direct programming effects in offspring are observed to exist (30).

### Oxidative Stress and Its Impact on Cardiovascular Complications

Obesity also contributes to physiological stresses like oxidative and endoplasmic stresses thereby leading to cardiovascular dysfunction and other complications such as increased risk of hypertension and gestational diabetes in which oxidative stress (OS) is a contributing factor (48, 49). A study (48) was conducted in pregnant women with normal prepregnancy BMI (*n* = 30) and those suffering from obesity (*n* = 15). Placental expression of an antioxidant marker, glutathione peroxidase 4 (GPx4) was measured. Typically, lower levels of GPx4 are associated with obesity. It was indeed found to be lower in the placenta of obesogenic mothers compared to non-obese women. Their study showed a direct relationship between lower GPx4 expression in the placentas of obese mothers, suggesting that such an obesogenic effect could be programmed into the offspring through the placenta. More importantly, adiposity in offspring of obese mothers can activate OS in the later stage of life ultimately causing cardiovascular complications (50). Hence, OS is one of several possible factors that could induce hypertensive disorders and neonatal diseases (51) with obesity.

The beneficial effect of maternal weight loss on offspring programming has been investigated (52). In this study, 172 children born to 113 obese mothers who experienced significant weight loss after biliopancreatic bypass surgery were compared to their siblings born before the mother had bariatric surgery. Average BMI's of the mothers before and after surgery were 48 ± 8 and 31 ± 9 kg/m^2^, respectively. The risk of future obesity was significantly lowered by 45% among children born following surgery.

### Maternal Obesity and Its Impact on Breast Milk

Maternal obesity has also been shown to cause differences in the composition of breast milk (53). In a study of 986 women and children pairs, mothers that were overweight or obese (OW/OB) had higher levels of obesity-promoting fatty acids (such as palmitic acid, adrenic acid, and dihomo-gamma-linolenic acid) and reduced protective fatty acids (such as docosahexaenoic acid, docosapenataenoic acid) in their breast milk. Higher percentiles for BMI were observed in infants born to these women, indicating a positive association between GWG and increased infant birth weight and parity (32). Further, a significant association was found between long-chain polyunsaturated fatty acid (PUFA) driven factor 1 in milk and higher weight-for-length, lower length-for-age, and lower head circumference-for-age of infants, suggesting altered composition of milk in OW/OB mothers to be a contributing factor to early growth in infants.

### Role of Nutritional Intervention

With the understanding of factors affecting maternal obesity and its implications on offspring, this section reviews the impact of various nutritional interventions on maternal obesity in humans. Nutritional as well as lifestyle (especially physical activity) interventions have been proven to be an effective measure to control GWG and reduce the negative effect of maternal obesity on offspring health.

A meta-data analysis performed by Thangaratinam et al. (54) suggested dietary intervention to be the most effective way to combat maternal obesity. Based on 34 different randomized trials, average reduction in maternal GWG because of the dietary intervention was 3.84 kg compared to 1.42 kg obtained through interventions like physical activity. An additional advantage of the dietary intervention was a significant reduction in the risk of preeclampsia. However, no evidence of decreases in cesarean section (C-section) rates were observed as a result of dietary intervention in their study. A randomized controlled trial of combined diet and capsule (either probiotic or placebo) intervention in a group of 230 pregnant women with prepregnancy BMI > 30 but without diabetes was performed by Okesene-Gafa et al. (55). Total GWG in the probiotic group was 9.7 kg as compared to 11.4 kg in the placebo group. However, average infant birth weights were comparable between the groups. This suggests that the probiotic intervention during this critical window is related to overall health improvement of only the mother, which in turn can ensure better health in the future for the offspring.

One of the nutritional supplements extensively studied is omega-3 polyunsaturated fatty acids (n-3 PUFAs). Monthe-Dreze et al. discovered that dietary inadequacy in n-3 PUFAs is associated with maternal obesity (56). They identified that the n-6/n-3 ratio (which is a marker for inflammatory status) was higher in obese women compared to normal pregnant women. Further, n-3 PUFA supplementation during 19–22 weeks of gestation until delivery illustrated that the obese women had an attenuated n-6/n-3 ratio that was 65% lower relative to women with normal BMI. Interestingly, these women also presented an attenuated response to n-3 supplementation compared to lean women. Overall, this study shows an association of higher inflammation status and reduced n-3 PUFA levels with maternal obesity. Maternal n-3 PUFA levels may influence overall growth and adiposity of offspring. Vidakovic et al. (57) conducted a prospective cohort study of 4,830 mothers and children to detect differences in overall growth and adiposity of children with regards to maternal PUFA levels during pregnancy. They measured plasma n-3/n-6 ratio during the second trimester of pregnancy along with body fat content in the child at 6 years of age. They established a strong association between lower n-3 PUFA and higher n-6 PUFA concentration during the second trimester with higher body and abdominal fat in childhood.

Human studies designed to comprehend the role of intervention on maternal obesity offers several advantages. They offer information on the direct impact of dietary or physical activity intervention on changes in body composition (fat), cardiovascular functioning, blood hormone levels, and so on. However, the major challenges in human studies are the long gestation period, difficulty in following up with the mother–child for long periods, which would offer a better understanding of the lasting effect as well as the influence of uncontrolled variables like environmental factors. Further, mechanistic studies to understand interventions at organ level, are extremely challenging and expensive in human subjects. In animal models such drawbacks are counteracted as they have shorter gestation time and are grown in a controlled environment throughout the studies to avoid potential errors with internal validity and confounding variables. A litter size of 8–12 pups in rodents at each pregnancy is especially convenient to achieve an understanding of various interventional strategies. Further, mechanistic studies to understand the effect of interventions in internal organs is easier to perform in animal models. Although the findings from the animal studies cannot be directly translated to humans to address obesity, the odds in animal studies being in favor of the benefits gained encourage researchers to opt for animal models.

### Animal Models for Maternal Obesity

Several different animal models have been utilized to understand the complex biological mechanisms of developmental programming in maternal obesity. Multiple considerations must be taken into account when choosing an animal, including but not limited to: similarity to humans (or the species that is being investigated), the research questions being pursued, ease of availability, and difficulty of care. Although it is more suitable to study primates who experience a similar pregnancy to humans, their gestational time is longer, which leads to slower research and higher overall costs (58). Adult mice, rats, pigs, and sheep are some of the most common animal models that have been used. Many of the commonly used animal models are predicated upon the fact that they have commonalities between their genetic origins and humans and in which the results drawn from these experiments can be extended to humans. A majority of these studies have been performed in rodent models owing to their reduced gestation period (~3 weeks) and maturity interval (~5 weeks).

In a mice study, females were fed either a high-fat diet (HFD) or chow diet 4 weeks prior to mating (59). Inflammatory markers were measured in fetal subcutaneous adipose tissue after 17 days of gestation. Offspring of the HFD group demonstrated higher mRNA levels of chemokine receptor-2 and tumor necrosis factor-α (TNF-α) and reduced levels of glucose transporter-4 (GLUT4), which indicates higher risk of developing obesity and metabolic disorders during adolescence. Corroborating with the previous study, Elhai et al. (60) performed a study in mice where dams consumed either a HFD or control diet (CD) during pregnancy and their offspring were then fed either a HFD or CD after weaning to see the relative differences between additional exposure to HFD or switching to CD. They observed that the HFD-fed offspring of both CD and HFD dams (C-H and H-H) as well as CD-fed offspring of HFD dams (H-C) demonstrated higher body weight, body fat, serum cholesterol, and blood pressure compared to CD-fed offspring of CD dams (C-C). This clearly demonstrates an attenuation of metabolic health after long-term exposure to HFD.

Another study by Wang et al. indicated that maternal obesity is one of the primary cause of altering genes that regulate cardiac function (61) in the fetus. They measured the cardioprotective marker (AMP-activated protein kinase; AMPK) in a sheep model; and identified significantly lower levels of phosphorylated AMPK in fetal hearts of the obesogenic group. Further, lower phosphorylation levels of c-Jun N-terminal kinase (JNK), insulin receptor substrate-1 (IRS-1), and PI3K-Akt was found in the fetal heart of offspring born to obese dams illustrating an alteration in cardiac insulin signaling, which directly induces IR and cardiac dysfunction in later stages of offspring life (61). In corroboration with this work, another study in the sheep model by Zhu et al. (62) investigated outcomes in terms of signaling pathways for maternal obesity programming in offspring. Obesity was induced by administering higher diet intake [50% more intake than the recommendation from the National Research Council (NRC)] in sheep (OB) and 100% of NRC recommendation (control; CD) to sheep from 60 days before conception to 75 days after conception. Reduced phosphorylation of AMPK was found in OB sheep compared to CD sheep, indicating reduced muscle development. These studies demonstrate the effects of maternal obesity on offspring skeletal muscle and cardiac development.

Loche et al. (63) investigated the effect of maternal and postnatal obesogenic diet on cardiac structure and function in mice. Offspring of both obese and non-obese dams were fed either a CD (7% simple sugar, 3% fat, and 15% protein) or HFD (10% simple sugars, 20% animal fat, and 23% protein) starting from 3 weeks of birth for 5 weeks. Heart weight, levels of transcriptional re-activators of cardiac fetal genes, contractile function genes as well as hypertension were measured in offspring heart at 8 weeks of age. Consumption of HFD led to a 23% higher offspring heart weight than CD offspring as well as increased frequency of hypertension. Additionally, the study found that the obesity status of the mother, by itself, was associated with both the modification of cardiac structure as well as increased cardiomyocyte area in offspring. Further, increased expression of cardiac fetal genes including contractile and matrix remodeling genes in offspring was also directly associated with maternal obesity providing direct evidence for the effect of mother's obesity in offspring cardiac health.

A similar study evaluated the differences in cardiac structure and function in offspring of obese vs. lean dams (64). Cardiac development in minipig offspring was studied at 3, 6, and 12 months of age by using echocardiography and histology after their mothers were supplemented with HFD or normal diet during pregnancy. They examined glucose metabolism through positron emission tomography (PET) and myocardial enzymes by mass spectrometry in the offspring. Offspring born to the HFD-fed obese mothers demonstrated higher left ventricular mass (+100%), larger stroke volume (+75%) as well as more cardiomyocyte nuclei (+28%) compared to their counterparts on CD. Additionally, higher levels of OS enzymes and myocardial IR were observed in the HFD-fed offspring along with elevated heart rate later in their adulthood. Their study sheds light into the lesser-known effects of maternal obesity on cardiovascular health of offspring. These studies provide direct comparison of the modification in phenotypes including organ size, fat content, etc., due to maternal obesity and thereby offer an overall mechanistic explanation that can serve as a basis for future human studies.

### Maternal Obesity and Impact on Appetite and Neuroendocrine System

Some of the alterations observed in terms of appetite and activity in offspring are due to consequences of programming effects on the brain (65, 66). Leptin and ghrelin are suggested to be associated in mediating metabolic malprogramming in the fetus due to maternal overnutrition (67). Higher amount of leptin and ghrelin cause permanent damage on hypothalamic function ultimately causing hyperphagia and obesity in offspring (68). This was demonstrated in mice by Samuelsson et al. (66), in which the dams were fed either a chow diet (7% simple sugars, 3% fat, 50% polysaccharide, and 15% protein) or an obesogenic diet (10% simple sugars, 20% animal lard, 28% polysaccharide, and 23% protein) starting from 6 weeks before mating through pregnancy and lactation. Leptin concentration in offspring born to obese dams was significantly higher as compared to the non-obese counterparts at 3 and 6 months of age. Moreover, offspring fed with HFD had higher daily energy intake illustrating development of hyperphagia at 4–6 weeks of age suggesting a potential alteration in the hypothalamus, a key organ that regulates appetite (65). Similarly, other studies also show that leptin and ghrelin in mother play a major role in assisting the growth of hypothalamic neural circuits during the neonatal period of offspring, and the implications of these factors in the lifelong metabolic alteration in the offspring (67, 69–71).

Although it is difficult to access the role of maternal obesity in the neurobehavior of offspring in human subjects due to several confounding variables, there has been significant research in animal models. Maternal obesity is found to increase the risk for neurodevelopmental disorders in offspring (72). The status in obese mothers which includes increased hormonal levels associated with obesity, availability of increased nutrition and higher levels of inflammatory factors are observed to induce effects such as neuroendocrine system disorders and delayed brain development through maternal programming in offspring. Further a recent meta-analysis by Menting et al. (73) showed that offspring born to obese mother are more likely to have higher locomotor activity as well as anxiety as compared to the non-obese counterparts.

### Maternal Obesity and Impact on Liver Function

Thompson et al. studied the effect of maternal HFD during pregnancy and lactation on liver histopathology and fibrosis in mice (74). Higher hepatocyte proliferation and stellate cell activation was observed in the liver of offspring born to HFD dams compared to offspring of CD dams at 12 weeks of age. Further, hepatosteatosis was higher in offspring born to HFD-fed dams and who continued on HFD postnatally. The exposure to HFD was found to be directly associated with non-alcoholic fatty liver disease (NAFLD) in adulthood.

In summary, studies in animal models have demonstrated that HFD-fed pregnant mothers give birth to offspring with phenotypes of increased adiposity, elevated blood pressure, higher body weight, and lower insulin sensitivity in offspring (75–77). Such phenotypes in animals align with observations of children born to insulin resistant women (78, 79).

## Nutritional Intervention in Animal Models

Mechanistic studies were performed using animal models to understand the effect of nutritional supplementation on maternal obesity and (80) offspring health during the early stage of (postnatal) life (81). Fish oil (FO) supplementation in dams during pregnancy prevented obesity and IR in adult male offspring of Sprague-Dawley rat in a study conducted in Auckland, NZ (80). In this study, female rats were divided into four groups: (i) Control diet (Con; 15% fat), (ii) Control diet supplemented with fish oil (Con-FO), (iii) High fat (HF) (45% fat) diet, and (iv) HFD with fish oil (HF-FO). With the corresponding diet fed throughout pregnancy and during lactation (until weaning), male offspring continued on CD until adulthood. Insulin sensitivity in HF-FO offspring was enhanced compared to HF-fed offspring, suggesting a direct association of FO consumption during pregnancy on the slowdown of IR in male offspring. This study highlights the need for further research on enhancing the overall metabolism of HF-FO offspring with proper dietary intervention.

A similar study demonstrated the comparative effect of maternal and postnatal FO supplementation on offspring metabolic health in a mouse model (81). Dams were fed HFD with FO (HF-FO) or without (HF) or a low-fat diet (LF). Diets were supplemented 8 weeks pregestation until the termination of lactation in offspring. After weaning, each set of the male offspring (F1) of HF-FO and HF dams were further divided into two groups: one continued on the same diet as mother (i.e., HF-HF and FO-FO) and the other group was switched and fed the opposite diet to the mother (i.e., HF-FO and FO-HF), respectively. Male offspring from each of the four sub-categories were compared with offspring from LF dams. They investigated relative changes in offspring phenotype and biological mechanisms among the different sub-categories and demonstrated that the continuous supplementation of FO prevented obesity-induced fat pad growth and retarded the expression of fatty acid synthase (FAS) as well as reduced IR. This study also showed that supplementation of FO can reduce liver inflammation by reducing cytokine expression. Taken together, all these studies call for the need to better define HFD during pregnancy in terms of essential and non-essential fats.

High consumption of saturated fat contributes to obesity while that of certain essential fatty acids, including PUFAs like alpha linolenic acid (ALA) (82), promote fatty acid oxidation, thus blunting obesity and its associated MS, especially IR (83, 84). Accordingly, supplementation of essential fatty acids during maternal obesity has been proven to attenuate metabolic dysfunction in offspring (85). Martin et al. (85) performed a study in a mice model involving an obesogenic diet with or without supplementation of sunflower oil and flaxseed powder that contained n-6/n-3 PUFAs in the ratio of 1.2:1.0. Upon weaning, a commercial diet was fed to all offspring and glucose homeostasis, body fat content as well as glucose-induced insulin secretion (GIIS) were assessed at 13 weeks in the offspring. They observed that offspring in the maternal flaxseed diet supplementation group demonstrated lower obesity rate, reduced IR, and counter balanced GIIS impairment compared to obesogenic diet.

Another study by Shomonov-Wagner et al. (86) also reinforced the effect of ALA in attenuating the IR associated to maternal obesity in adult mice. In their experiment, female mice were fed either a regular diet or an obesogenic diet rich in saturated fatty acids (SFA). Dams fed SFA's were further divided into two groups based on a partial substitution of SFA with ALA, with diets maintained throughout pregnancy and lactation. Offspring born to SFA-fed obese mice demonstrated higher hepatic lipid content and increased desaturase activities. Partial substitution of ALA was found to prevent lipid accumulation and significantly decrease HOMA index and desaturase activities.

While most studies in rodents involve diet-induced obesogenic effects, alteration in the chemistry of a high-calorie (HC) obesogenic diet itself can affect offspring metabolic health. A study compared modified fatty acid (HC diet of equivalent energy but with large proportion of medium-chain fatty acids and lower ratio of n-6/ n-3 PUFAs to regular HC diet (87). Lower GWG and reduced hepatic fat accumulation were observed in the modified fatty acid diet group compared to the regular HC diet. Genes involved in hepatic *de novo* lipogenesis were expressed at lower levels in the modified fatty acid group, revealing potential mechanisms of reduced hepatic fat accumulation. This study provides a framework to design better diets for healthier pregnancies.

Another example of nutritional supplementation in maternal obesity prevention was recently demonstrated by Maragkoudaki et al. They demonstrated the role of prebiotic polydextrose (PDX) supplementation in preventing maternal obesity and the consequences of programming in the offspring (88). Female mice were fed either a CD or an obesogenic diet for 6 weeks prior to mating. Within the obesogenic dams, half of the mice were supplemented with 5% PDX in drinking water throughout pregnancy and lactation. To determine the impact of PDX supplementation, calorimetry was used to quantify energy intake and expenditure in offspring at 3 and 6 months of age. Offspring of obese dams showed increased body weight and higher white adipose tissue (WAT) mass compared to their non-obese counterparts. Offspring of PDX supplemented obese dams had lower body weight and WAT mass compared to offspring of obesogenic dams who consumed HFD. Further, PDX supplementation increased night-time energy expenditure as compared to offspring of obesogenic dams. One of the mechanisms indicated for this increased expenditure was an increase in the skeletal muscle mtDNA copy number.

An additional nutritional supplement that has demonstrated a potential role in offspring cardiovascular health is N-acetyl cysteine (NAC) (89). Supplementation of NAC in diet of pregnant obese mice reduced ventricular hypertrophy in offspring. Further, higher expression of cardiac fibrosis markers was found in the heart of neonatal male mice born to obese mothers (M-OB), which ultimately led to left ventricular hypertrophy as early as 1 week after birth. M-OB offspring had increased heart weight and higher interventricular septal thickness compared to the lean counterparts. Meanwhile, offspring born to the NAC treated obese dams M-OB/NAC had lower heart weight but comparable ventricular hypertrophy. Their study also illustrates reduced OS with NAC revealing potential mechanisms of NAC for ventricular hypertrophy in offspring of obese dams.

In summary, nutritional interventional studies in animals help us comprehend the potential beneficial mechanisms of bioactives in offspring. Insights from these studies justify the need for nutritional intervention studies in humans to prevent obesity and associated metabolic dysfunction.

## Physical Activity During Pregnancy

Apart from nutritional interventions, physical activity is another parameter known to reduce maternal obesity and its complications. The American College of Obstetricians and Gynecologists (ACOG) recommends 150 min of moderate intensity exercise every week for pregnant women, with around 30 min of exercise each day (90). American College of Obstetricians and Gynecologists recommends that pregnant women continue their physical activity through all three trimesters if their health and pregnancy are normal and no complications exist (91). Though the benefits of physical activity during pregnancy are well-established, as of 2010, only 15% of pregnant women met the physical activity guidelines of 150 min a week (92). This could be because physiological changes occurring during pregnancy could contribute to lack of physical activity (93). Moreover, culturally, pregnant women have been discouraged from physical activity to avoid miscarriage in the first trimester (94). Even in the 1990's, intense physical activity was not prescribed during gestation (95). Now, there is abundant data regarding benefits of physical activity that include reducing risk of preterm labor or miscarriage or postnatal fetal demise. Here, we review the beneficial effects of physical activity in both humans and animal studies (Table 1).

**Table 1:** List of animal studies analyzing the beneficial effects of physical activity.

**Dam**			**Graphical representation of the study**	**Offspring**		**References**
**Diet and duration**	**Exercise**	**Exercise duration**		**Sex and (Species)**	**Results**	
Chow (16.7% fat) 10 days PG to PND 21	RW	10 days PG—PND 12	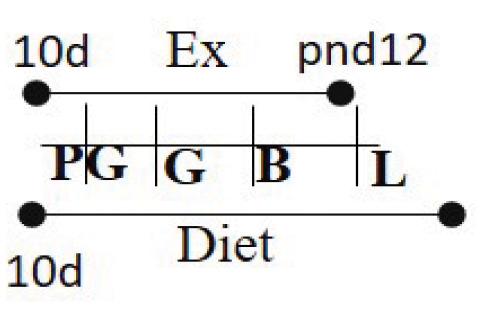	M and F (R)	Increased glucose uptake in skeletal muscle in offspring of exercised dams compared to offspring of sedentary dams	(96)
Chow 16.7% fat (same as exercise duration)	RW	≥7 days prior to G—PND 14	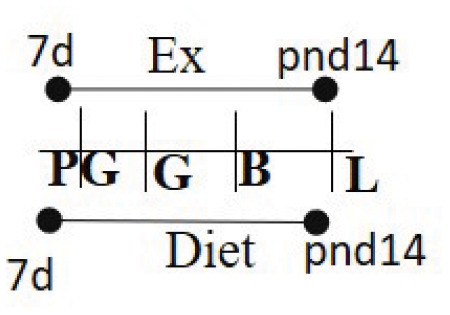	M and F (M)	Increased insulin sensitivity among the exercised cohort.	(97)
Chow 21% fat or HFD 60% fat 14 days PG—G	RW	14 days PG—B	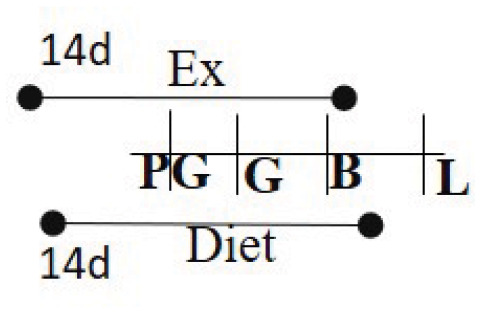	M (M)	Increased glucose metabolism among exercised cohort.	(98)
HFD 26% fat or Chow 5% fat Day 21 of life—L	RW	Day 90 of life—B	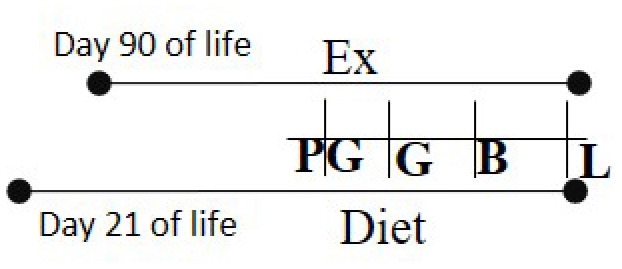	M and F (R)	Exercised cohorts showed lower triglyceride/glucose/oxidative stress levels	(99)
Chow or HFD (60%) 42 days before PG—B	RW	42 days before PG—B	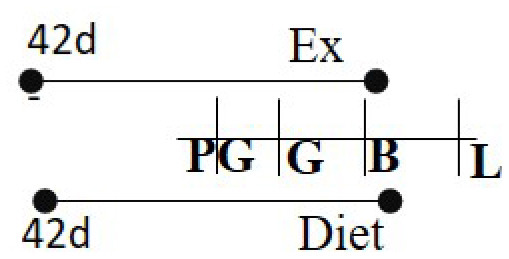	M and F (M)	Pgc-1α was hypomethylated with higher Pgc-1α levels in exercised cohorts.	(100)
HFD 60% fat 42–49 days of age—PND 21	RW	14 days prior to G—PND 21	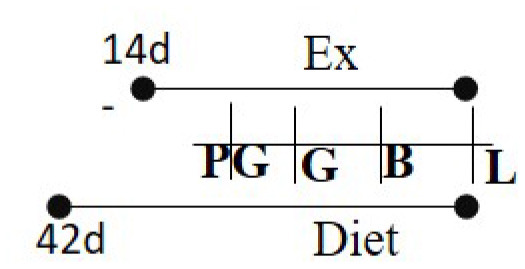	M (M)	Beta cell mass and size was much smaller in offspring born to exercised dams.	(101)
Chow or HFD-2 types: 43, 59% 42 days prior G-B	RW	10 days prior G—B	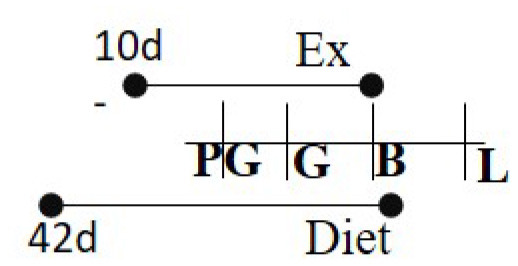	M and F (R)	Expression of GLUT4 and PGC-1α levels were upregulated among the exercised cohorts in skeletal muscle.	(102)
Chow 21% or HFD 60% 14 days PG to L	RW	14 days prior to G—B	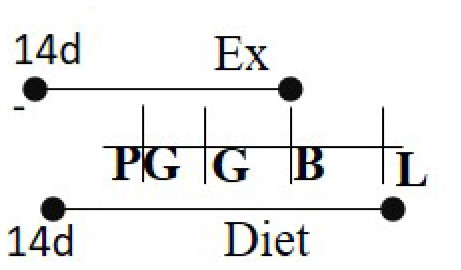	M and F (M)	Exercised dams presented lower rates of hepatic glucose production and liver triglycerides. Males tended to have higher overall levels.	(103)
Chow diet 3% or 20% lard diet Starting at 3 weeks of age for 15 weeks	TM	One week prior to G—G day 17 (20 min × 5 days/week		M (M)	Lower cardiac hypertrophy and dysfunction in offspring of exercised dams.	(104)
Chow (16.7% fat) 10 days PG to PND 21	TM	14 days PG-L	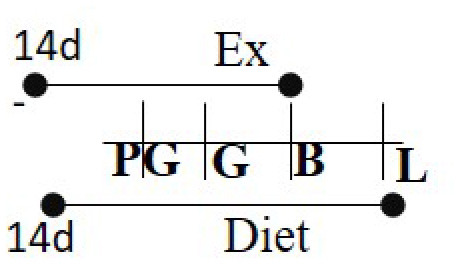	M and F (R)	Exercising cohorts showed lower fat accumulation, plasma insulin, and glucose levels.	(105)
Chow 16.7% fat (same as exercise duration)	RW	63 days PG-L	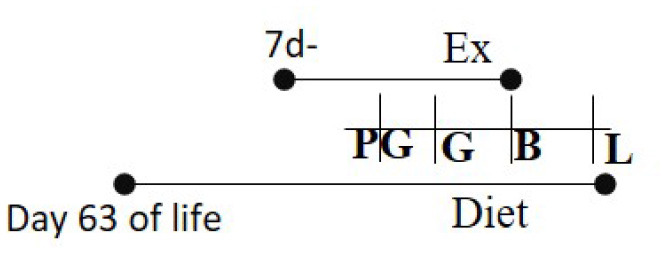	M and F (M)	Elevated levels of AMPK and PGC1a and reduced hepatic lipogenesis in liver among exercise invention group.	(106)

*RW, Running wheel; TM, Treadmill; HFD, High-fat diet; M, males; F, Females; PG, pregestation; G, Gestation; B, Birth; L, Lactation; PND, Postnatal day*.

### Physical Activity and Its Role in Reducing Adverse Pregnancy Complications

Physical activity during pregnancy provides gestational weight control and reduces pregnancy complications like preeclampsia and GDM (106). Studies have demonstrated that physical activity improves labor experience and reduces its complications (20, 21). In line with this, increased physical activity during pregnancy is negatively correlated with Pitocin use during labor, emergency interventions during delivery, and C-sections (107). Further, women who were physically active were twice as likely to dilate from 4 to 10 cm in under 4 h during labor, as opposed to those with minimal or no physical activity, thus leading to a better labor experience. Likewise, physical activity may assist in reducing the risk of adverse delivery outcomes resulting from maternal obesity, such as shoulder dystocia, miscarriage, and postpartum hemorrhage (20, 21). Lastly, physical activity also decreases total postpartum recovery time (107). Hence, achieving recommended levels of physical activity is certainly beneficial to the pregnant mother (28).

Physical activity has become an imperative guideline for optimal outcomes for both mother and the offspring. A 2020 study known as the Born in Bradford study, followed 7,305 mother-child pairs of either Pakistani or white-British origin and assessed the relationships between physical activity levels and several metabolic markers: fasting glucose, post-prandial glucose, triglycerides, and the amount of adipose tissue in women and their children at birth. At 26–28 weeks gestation, participants were divided into four groups: inactive, somewhat active, moderately active, and active (≥150 min of moderate intensity physical activity per week). They found more than half of the participants were inactive during their second trimester. Total amount of adipose (measured via triceps skinfold and mid-upper arm circumference) was significantly lower in the active group compared to the inactive group for white-British women. Additionally, metabolic results from 26 to 28 weeks of gestation showed that white-British women in the active group had significantly lower fasting insulin and post-prandial glucose compared to the inactive group. Total triglycerides were significantly lower in the active and moderately active groups. Total skinfolds, both triceps and subscapular measurements, were significantly lower in the active and moderately active groups. In the Pakistani group, women in the somewhat active group had significantly lower systolic blood pressure, lower triglycerides and higher HDL compared to the inactive group. Total skinfold measurements were not significant, though it was decreased in the moderately active and active Pakistani groups. The smaller sample size may explain the lack of significant findings in those categories. However, GWG and offspring birthweight was comparable between the groups. This study establishes further prerequisite for physical activity during pregnancy as it may help attenuate insulin, glucose, and lipid markers. Additionally, the study shows minimal risk of adverse effects on birth weight or gestational age from physical activity. Dose-dependent relationships also existed between physical activity and offspring arm and abdominal circumference, suggesting better neonatal outcomes from maternal physical activity (108).

### Physical Activity and Birthweight/Birth Outcome

Physical activity also has positive implications for reducing risk of preterm birth. A 1983 case–control study on 175 women with preterm births and 331 women with full-term births showed that those who performed either sports or general physical activity during pregnancy had a significantly lower risk of preterm births than their less active counterparts (109). Similarly, a larger case–control study of about 7,000 participants indicated that maternal exercise reduced the risk of preterm birth. However, the association was found only when the exercise was <150 min/week (110). Exercise totaling >150 min/week did not reduce this risk. In addition, a 1984 epidemiological study compared women participating in an endurance-based exercise regimen through their third trimester to women who ceased exercise prior to 28 weeks. Women who exercised delivered 8 days earlier and their offspring weighed 500 g less than women who ceased exercise before 28 weeks (111). Additionally, a 2018 study showed that a lack of physical activity during pregnancy is a risk factor for macrosomia (112). Finally, a study that measured activity in terms of energy expenditure (low moderate activity: <1,000 kcal/week; heavy activity >1,000 kcal/week) and identified that the higher activity group had decreased risk of preterm delivery and a shorter duration of labor than their counterparts (113). However, a 1990 review suggests that the proposed negative association between physical activity and preterm birth may be strongly influenced by socioeconomic status and the amount of time spent standing throughout the day. Further, infants from sedentary, active or standing groups did not differ in preterm delivery outcome. The study did corroborate with other data, further proving that women who are physically active during pregnancy do not have a heightened risk of preterm delivery (114). Physical activity during pregnancy is therefore low risk and high benefit with respect to birth outcomes, birthweight, and course of labor and delivery.

### Physical Activity and GDM

An important undeniable outcome of maternal exercise is lowering the adverse effect of GDM (115). Pregnancy causes physiological and biochemical changes that can contribute to a higher likelihood of IR. Exercising several times a week led to positive outcomes for both mother and baby in the following studies. A possible mechanism for decreased GDM in pregnant women who incorporate physical activity is better glucose control leading to better insulin sensitivity and glucose uptake (116). A study conducted in 300 Chinese women identified that those who exercised (supervised cycling three times a week for 30 min) had lower incidence of GDM (117). A potential reason for the reduced rates of GDM could be that the exercise group had less GWG by 25 weeks of gestation. Offspring born to the exercise group had significantly lower birth weight compared to pregnant women who did not exercise. This is an important outcome considering that obese mothers are at higher risk of delivering a higher birth weight infant (macrosomia). This corroborates with a study showing that exercise, along with dietary and lifestyle counseling between gestational weeks 8–12 reduced GDM incidence (118). Coincidently, women who were overweight with GDM and performed resistance training had reduced need of insulin compared to women who did not exercise (119). Lastly, another study demonstrated that the progression of GDM to T2D among postpartum women was reduced in those who followed the recommended guidelines of physical activity (115). However, this study looked at overall lifestyle interventions and physical activity was only one of the parameters (120).

### Physical Activity and Maternal Weight Gain

Much of the adverse effects witnessed during or after pregnancy are related to excessive GWG (11, 22). These adverse effects include, but are not limited to preeclampsia, labor complications, increased C-section rates, macrosomia, maternal weight retention postpartum, and increased offspring BMI throughout the life course (17, 121). In a meta-analysis of 12 studies comparing physical activity levels with GWG, it was found that total GWG was lower in the exercise group in 7 of the 12 studies (11). A 2012 study investigated the relationship between physical activity and GWG in a Chinese cohort of 862 women in their second trimester. Participants received a pedometer and were categorized into four activity groups based on their step counts: sedentary (<5,000 steps), low active (5,000–7,500 steps), somewhat active (7,500–10,000 steps), and active (≥10,000 steps). By the end of their pregnancies, the active group had gained 1.45 kg less than the sedentary group, suggesting that physical activity has an influential role in GWG. The adjusted OR for excessive GWG in the second trimester was 0.59. Results of the study corroborate with other research showing a negative association between physical activity and excessive GWG (122). Similarly, the aforementioned 1984 epidemiological study showed that women who continued their endurance-based physical activity regimen from preconception ended up gaining 4.6 kg less than their counterparts who ceased exercise 28 weeks before gestation (123). Though most of the trials performed in women have demonstrated that exercise has beneficial effects on weight gain or offspring health outcomes (121, 124, 125), the UPBEAT trial conducted in the UK in about 1,600 women with BMI > 30, demonstrated no effect on weight gain [19]. Some studies show reduced GWG in obese pregnant women, while other studies showed no difference in GWG or birth weight when women performed physical activity. Overall, physical activity during pregnancy has been consistently associated with decreased GWG, but more trials are needed to investigate the effect of physical activity on biomarkers such as serum triglycerides and insulin secretion.

### Aerobic Exercise, Resistance Training, and Pregnancy

Guidelines suggest that if a woman was sedentary prior to pregnancy, a progressively increase in physical activity during pregnancy could be beneficial. Likewise, if the mother was previously active, then continuation of their established activity is recommended. There is no established safe upper level of exercise intensity. Up to 40 min per day of moderate intensity is beneficial for low-risk pregnant women, and the safest activities are walking, strength training, and water-based exercises. Contraindications include severe anemia, multigravida, incompetent cervix, restrictive lung disease, placenta previa after 26 weeks, or vaginal bleeding of unknown cause in the second and third trimesters (107).

Studies evaluating resistance training in pregnant women have shown mostly positive results (126). A 2009 study demonstrated that resistance training during the second and third trimesters of pregnancy did not alter the type of delivery nor infant outcome compared to pregnant women who did aerobic exercise (111). In line with this, a study by Bgeginski showed that resistance training during pregnancy did not alter fetal heart rate (FHR) when tested at 22–36 weeks of gestation. Thus, resistance training does not appear to pose any risks to the fetus throughout the second and third trimesters (127). However, these studies also demonstrated that adverse outcomes may stem from any exercises performed while in the supine position during pregnancy (128, 129). This is in line with another study that showed resistance training during the third trimester increased FHR of the offspring, specifically in the supine position. These data suggest that supine exercises such as core exercises are better avoided during pregnancy. In contrast, a recent systematic review indicated lower incidence for such adverse effects (117, 130). Even though exercise is beneficial, certain alterations should be made in terms of the intensity and duration of resistance exercise because of the physiological and anatomical changes that occur during pregnancy.

Apart from resistance training, aerobic exercise was also studied for its relationship with gestational hypertensive disorders. In a systematic review, the relative risks of gestational hypertension and preeclampsia with exercise were measured in 5,075 women participating in 17 randomized controlled trials. On average, women completed 30–60 min of aerobic exercise 2–7 times each week. Seven of the studies demonstrated that the relative risks of gestational hypertension and preeclampsia was lower among women participating in aerobic exercise. The exercise group had a 2.5% incidence of gestational hypertension compared to 4.6% in the control. Interestingly, the incidence of preeclampsia was similar between groups (2.3% compared to 2.8%, respectively). Cesarean deliveries also decreased by 16% in the exercise group (93).

Another study found that hypertension was reduced in pregnant women who performed circuit (resistance plus aerobic exercises in one session) training compared to pregnant women who performed aerobic exercise only. However, no difference in gestational age or other infant outcomes were found (131). Interestingly, women who underwent circuit training had lower rates of C-sections and faster postpartum recovery times following delivery (132). Prior studies indicate that women who perform continuous exercise during the third trimester significantly reduced the FHR, which may be due to stress on the fetus suggesting that continuous exercise during the third trimester may lead to adverse outcomes (133). Importantly, the risk of musculoskeletal injuries is higher in pregnant women than non-pregnant women, which may be due to higher secretion of the hormone relaxin, a hormone intended to widen the cervix and relax ligaments around the pelvis but could have systemic effects on connective tissue. Common issues during pregnancy that are related to the effects of relaxin include pain in the pelvic region, which has the potential to decrease function and increase chances of injury during physical activity (134). In summary, lower intensity exercise seems to cause no difference in FHR, while few studies show it may be beneficial. However, high-intensity exercise >20 min is not recommended. More evidence is needed to better understand the risks and benefits of strength training during pregnancy.

### Maternal Exercise and Its Impact on Cardiovascular Outcomes

The NeoETIP (Neonates in the Exercise Training in Pregnancy) trial followed obese and overweight women. In all, 55 women participated 15 were obese and 4 were overweight (135). The women in the exercise group performed moderate intensity exercise weekly from 14 weeks until delivery. Neonatal cardiac function was measured via ECGs at 1–3 days old and 6–8 weeks old. Infants of obese women had impaired cardiac function and output at both timepoints. Infants born to mothers in the exercise group did not show any significant difference on cardiac function compared to the control group. More research with larger sample size is needed to better understand how maternal exercise impacts other cardiac functions in the offspring.

There are alterations or increases in cardiac output as pregnancy progresses, due in part to increases in total blood volume. Pregnant women who exercise are more likely to experience low resting heart rate (RHR) and have increased cardiac volume in response to exercise (136). An increased heart rate variability (HRV) is exhibited in women who are considered active because aerobic exercise improves parasympathetic control at rest, which correlates with better maternal and fetal outcomes (137). Hence, exercise could be a useful intervention to reduce hypertension in pregnant women. Further, pregnant women who exercised had infants with higher HRV compared to infants of women who did not exercise, suggesting that infantile cardiac output is improved in the exercise group (138). Thus, exercise during pregnancy influences the developing cardiac system.

Increased sympathetic activation is associated with pregnancy-induced hypertension, and preeclampsia is more likely in pregnant women with preexisting chronic diseases such as obesity and T2D. Research suggests that increased physical activity during pregnancy increases HRV (139–141). A low HRV denotes poor sympathetic control at rest, and a potential decrease in fetal cardiovascular adaptations postnatally. Fetal response to aerobic exercise includes development of fetal autonomic control within the cardiovascular system, which was inferred from decreased FHR and increased fetal HRV in response to maternal aerobic exercise at 36 weeks gestation (138). Additionally, fetal response to maternal resistance training has also been measured, though a minimal volume of research exists. A 2001 study showed that an increase in either intensity, frequency, or duration of resistance training was negatively correlated to adverse fetal outcomes (142). Further, non-continuous physical activity, such as weight lifting appears to be positively associated with fetal HRV (143, 144). Both aerobic and resistance exercise training do not exhibit adverse fetal cardiovascular outcomes. Though there appears to be ample data suggesting higher benefit and lower risk conferred by physical activity during pregnancy, more research is required to understand the sympathetic activation during exercise in pregnancy.

## Exercise Studies in Animal Models

Though human studies provide more generalizable results, interspecies research allows us to perform mechanistic studies that may not be extended spontaneously to humans and special populations. The types of exercise that are commonly employed in such studies consist of (voluntary or involuntary) wheel running, swimming, and resistance exercise. Factors such as intensities, frequencies, and duration can be altered to gain most accurate results of the study. In addition, exercise may be deemed short- or long-term, based on the length of the exercise regimen. Another important aspect to consider when selecting an exercise regimen for the animals is whether the exercise is performed voluntarily. The animal models must have the motivation or drive to exercise. Treadmill running has been a popular choice for many animal models since it is easy to measure, similar to wheel running (rotations/min) taken up by animals voluntarily as well. All the above criteria should be carefully considered and chosen to provide the most precise and relevant results for the study.

One study in rodents evaluated the association between voluntary maternal exercise during prepregnancy and pregnancy and offspring insulin and glucose sensitivity. Female rats were fed diets with 16.7% fat and either had access to a wheel (exercise group) or no access to it (sedentary group) within the cage (96). The intervention started 10 days prior to conception and lasted through pregnancy and until postnatal day 12 (the gestational length of mice is 21 days). Male and female offspring were weaned in sedentary cages for the duration of the study (15 months) to determine the influence of maternal exercise. As expected, offspring of dams that exercised weighed significantly less than offspring born to dams that were sedentary. Female offspring of exercised dams had higher glucose turnover compared to offspring born to sedentary dams at 10 months of age. Offspring born to exercised dams had higher glucose uptake in muscle with lower uptake in hearts than those born to dams that were sedentary, with no difference in glucose uptake in the adipose tissue. Further, these offspring had lower serum insulin levels indicating beneficial effects of maternal exercise. This study concluded that the offspring born to dams that exercised were less susceptible to T2D and that maternal exercise had a generational effect on offspring health. This study corroborated with another study showing that male and female offspring born to dams who had access to wheels during gestation had better glucose transport compared to dams that were sedentary, with greater differences observed in male offspring (97). This study also identified lower body fat mass with higher lean mass in male offspring born to exercised dams indicating lower risk of obesity. These results indicate higher insulin sensitivity and lower glucose tolerance with a lower probability to develop into T2D. However, these studies only tested the effect of a low-fat diet (16.5% fat, 56.8% carbohydrate, and 26.6% protein) in combination with voluntary exercise during prepregnancy and pregnancy.

Other studies have investigated the role of different diets along with the benefits of maternal exercise. Vega et al. studied the effect of exercise on maternal rats while consuming either a normal diet (control, 21% fat) or a HFD (60% fat) in preventing the adverse outcomes related to maternal obesity (99). The diet intervention and exercise regimen began at 3 months of age in rats and lasted throughout pregnancy. Control and HFD-fed rats were further subdivided by access or no access to an exercise wheel, thus, creating four groups. At 1 month of age, male offspring born to obese dams in the exercise group had lower triglycerides, leptin levels compared to those born to obese sedentary dams indicating the beneficial effects of exercise in reducing early obesity. Further, these parameters were comparable in control rats with or without exercise. However, the above markers were not altered in female offspring suggesting sex-dependent programming. It is possible that differences in both sexes may become evident with a longer duration follow-up.

An elegant study by Stanford et al. tracked the effects of timing of maternal exercise (98) on offspring health. Mice were fed either a chow diet (21% fat) or HFD (60% fat), then further subdivided to access an exercise wheel either only during pregestation (2 weeks prior to gestation) or during gestation. The last two groups had access to the wheel throughout gestation and all groups were compared to mice with no wheel in the cage. Male offspring were followed for 52 weeks. They found that mice of dams that had access to wheels during both pregestation and gestation had improved glucose tolerance and peripheral insulin sensitivity compared to all other groups.

Building upon these studies, a separate study investigated the role of exercise in maternal programming by comparing levels of methylated and total peroxisome proliferator-activated receptor-γ coactivator 1-α (Pgc-1α) between offspring born to dams that exercised and those were sedentary (100). Female dams were fed either a normal chow diet or HFD with no exercise and were compared to dams fed HFD and exercised (voluntary wheel running). Methylation was measured at birth and at 12 months in the offspring. Offspring born to exercised dams had lower levels of methylated Pgc-1α compared to offspring born to sedentary HFD dams, suggesting hypomethylation, a beneficial programming change. Further, exercise increased the levels of Pgc-1α, a marker that serves as a good indicator of skeletal muscle oxidative capacity. These data were consistent with increases in other downstream players of Pgc-1α, like Glut4, Cox4, and Cyt c. The improved oxidative capacity of the skeletal muscle was in line with improved glucose tolerance observed in offspring at 9 months of age born to exercised dams (100). Another study measured improvements in skeletal muscle in male and female offspring on day 19 after birth as a measure of beneficial effects of maternal exercise (102). Higher levels of Glut4 in skeletal muscle were observed in male and female offspring born to HFD dams engaged in exercise during pregnancy. Lastly, exercise also benefitted the mother by lowering triglycerides levels in dams that exercised vs. those only on HFD without any exercise.

Similar to improvements in skeletal muscle, exercise also improves the phenotype of pancreatic beta cells (101). Male offspring of obese dams that engaged in exercise had better glucose tolerance and improved beta cell size when measured at 52 weeks compared to offspring born to sedentary dams. Interestingly, the same study found an additive effect of paternal and maternal exercise on beta cell phenotype. This suggests that programming may be related to the paternal preconceptional health and behaviors as well as maternal health in this regard. Given these results in beta cells and epigenetic changes observed in skeletal muscle, it would be interesting to understand the methylation patterns of insulin-producing genes in pancreatic beta cells in offspring with respect to maternal as well as paternal exercise patterns.

Another organ involved in glucose homeostasis, the liver, has improved function in the offspring born to exercising dams. In one study, female dams were fed a chow diet (21% fat) or HFD (60% fat) and the groups were further subdivided to have exercise 2 weeks prior to, and through, gestation, or neither (103). Offspring were followed for 52 weeks. As expected, HFD negatively impacted glucose tolerance compared to mice fed a chow diet. Further, HFD reduced hepatic metabolic function that was measured by hepatic glucose output and by lower liver enzyme expression. These markers were improved in offspring born to obese dams that exercised compared to offspring born to HFD-fed dams without exercise. Overall, existing research suggests that maternal exercise improves metabolic function of all glucose homeostatic tissues under diet-induced obesogenic conditions. However, the studies found minimal additional benefits of exercise when mice were fed a standard or CD.

The studies presented here all used voluntary exercise, measured by wheels provided in cages. Few animal studies used involuntary exercises such as swimming or treadmill. In one such study, female dams were trained with forced swimming 60 min per day (moderate exercise load) for 6 weeks prior to mating (145). Offspring born to the dams were then fed HFD and remained sedentary to understand the effect of maternal exercise. They found that the offspring born to exercised dams had lower body weight, higher energy expenditure, higher adiponectin, and lower leptin levels compared to offspring born to sedentary dam. This study indicated the importance of maternal exercise as the offspring showed positive outcomes even with HFD challenge.

To understand the molecular mechanisms of physical activity, a study employed involuntary treadmill intervention, of 20 min per day from 1 week prior to conception up to gestational day 17 during pregnancy (104). Treadmill intervention improved offspring cardiac function and prevented cardiac hypertrophy at 8 months of age compared to offspring of sedentary dams, with there being no difference in hypertension. These results demonstrate the impact of maternal exercise on cardiovascular health in the offspring. Another study by Riberio et al. used a similar low-impact treadmill training regimen in the dams but tested the impact of exercise during pregnancy and lactation (30 min of treadmill running 3x per week at VO_2_ max of 30%) (146). Further, postnatal over nutrition was tested in mice by reducing the litter size of each dam (from 9 pups to 3). They observed that maternal exercise prevented the metabolic dysfunction in offspring that received postnatal overnutrition indicating that this could be in part due to alterations in composition of the breast milk. In line with this, breast milk from mothers suffering from obesity had altered fatty acid composition with lower n-3/n-6 ratio, which could induce obesity, compared to lean mothers.

As mentioned, there are many positive metabolic effects of physical activity during pregnancy, though specific mechanisms are still not well-understood. A 2020 study investigated a possible mechanism between gestational physical activity and offspring hepatic protection against NAFLD that could be caused from HFD (105). They posited that a healthy *in-utero* environment may provide protection from an obesogenic postnatal environment that may foster the development of NAFLD in adulthood. Maternal mice were divided into exercise (INT) and sedentary groups (CO) and maintained on a standard chow diet (CD) to purport a healthy gestational environment. The offspring either continued on CD or were placed on an HFD on postnatal day 70 and were monitored through postnatal day 112. Interestingly, the CO-HFD offspring increased body weight rapidly after postnatal day 70, and INT-HFD offspring maintained significantly lower body weight throughout the entire postnatal period. Liver samples were analyzed at postnatal day 112; CO-HFD mice showed signs of NAFLD and NASH (non-alcoholic steatohepatitis) after only 6 weeks on the HFD, whereas the INT-HFD mice did not exhibit these signs. Lastly, maternal physical activity had a large influence on CYP7A (cholesterol 7 alpha-hydroxylase), an enzyme relevant in cholesterol and bile metabolism and FAS. The INT-HFD had increased CYP7A and decreased FAS production, suggesting better liver function and a lower chance of developing NAFLD or NASH. Overall, INT-HFD offspring exhibited a significant protective effect compared to the CO-HFD, suggesting a possible mechanism that maternal exercise poses on offspring health.

A similar study also investigated possible biochemical mechanisms that could explain the observed protective effects of exercise on obese, maternal mice during gestation (147). Control maternal mice were on a CD and remaining maternal mice were fed an obesogenic diet and divided into sedentary and exercise groups. Exercise consisted of 20-min treadmill sessions five times a week for the first 17 days of gestation. Samples were taken at day 19 of gestation after dams were fed. Both obese groups weighed more than the control group, and sedentary obese dams had a greater proportion of WAT than the obese dams that exercised. Hepatic and skeletal adipose content was significantly greater in the obese groups compared to control, though they were not different from each other. This may be due to smaller sample size (control *n* = 6, obese-sedentary *n* = 6–7, obese-exercised *n* = 5) and short study length of only 19 days. Cholesterol and triglyceride levels did not vary between the groups. Plasma insulin varied significantly across all groups and was highest in the obese-sedentary group followed by the obese-exercised group. Finally, hepatic insulin receptor concentration analysis showed that obese-sedentary and obese-exercised dams had decreased receptor concentration compared to control, but only the latter group also had decreased MAPK, AKT, and p110β (an isoform of PI3, a kinase involved in insulin signaling). Larger changes were observed in the skeletal muscle of obese-exercised dams, specifically 4EBP (a eukaryotic translation initiation factor) which has implications in muscle protein synthesis. Finally, general insulin signaling through the MAPK/mTOR pathway, insulin receptor concentration, and Akt were decreased in the obese-sedentary dams but all were partially rescued in the obese-exercised dams. These findings suggest that exercise has tissue-specific and graded effects across the maternal body with majority of the implications in adipose tissue and skeletal muscle. Exercise may have an ability to restore insulin sensitivity to levels observed in a non-obese mouse without any associated damage to the developing fetus. These studies highlight the benefits of exercise on both maternal and offspring tissues during gestation and postnatally.

In a separate study in infant mice, the protective effect of maternal aquatic exercise from an obesogenic diet was assessed (33). Three-week-old maternal mice were put on either a CD (12% fat) or HFD (45% fat) and subsequently divided into CD-sedentary (CD-sed), CD-exercise, HFD-sedentary, and HFD-exercise for 13 weeks before conception and for the entire length of gestation. At delivery, all maternal mice transitioned to a CD. Interestingly, lactation was the sole energy source for all offspring. Four-week-old pups were randomly selected from each group for analysis. GTT and an ITT were administered 15 min after glucose/insulin injections and were significantly different between all offspring groups at postnatal day 28, with the highest blood glucose levels and body weights in the offspring of HFD-sed group. Free fatty acid, triglyceride, cholesterol, and IL-6 (a proinflammatory cytokine) levels were also measured. The HFD-sed group had the highest levels of IL-6, triglycerides, and cholesterol compared to both CD groups, with there being no difference in plasma FFA concentrations. These findings suggest that an HFD diet combined with a low-activity lifestyle establishes a foundation for obesity and other chronic diseases. Lastly, researchers measured digestive hormone concentrations and found that male offspring of the HFD-exercise group had significantly decreased glucagon and GIP secretion compared to the male pups from the HFD-sed group. The values of the HFD-sed group for these hormones were not different from the CD groups, suggesting that exercise was able to fully rescue the homeostatic levels of these hormones. Female offspring showed a similar effect; only glucagon in the HFD-sed group was greater when compared to the CD-sed group, but there was minimal improvement when compared to the HFD-exercise group. These alterations in hormone output between groups once again suggest better homeostatic regulation due to exercise. Higher energy output from increased physical activity decreases circulating levels of triglyceride and improves insulin sensitivity toward the available glucose and triglyceride. Sequestration of glucose and triglyceride for exercise helps mitigate over accumulation of triglyceride, which has ample implications in preventing disease states, and hence a better quality of life.

Exercise interventions in maternal HFD groups downregulated much of the adverse metabolic changes that were observed in maternal HFD-sedentary groups. Further, the type of exercise does not appear to be specific; significantly greater exercise observed in an active lifestyle modulates hormone production and glucose/triglyceride utilization to levels observed in maternal CD groups. In all studies, body weight was lower in HFD-exercise offspring compared to the HFD-sedentary counterparts, and some studies observed differences in body composition as well. Other studies also suggest sex-dependent programming on benefits of maternal exercise to the offspring. Mainly, male offspring were more responsive to metabolic improvements from exercise. This may be due to additional hormones present in female offspring and potentially different baseline body composition values geared toward successful reproduction. Though the majority of studies had different lengths and investigated maternal effects at varying lengths postnatally, the theme of exercise as a protective mechanism against adverse effects of maternal obesity remains consistent.

## Conclusions

Based on the review of research in maternal obesity, there is evidence toward the definitive role of maternal obesity and physical activity on the overall health and development of the offspring. Maternal diet becomes increasingly important throughout the lifespan and particularly during gestation, as the health status of the mother has implications for the efficacy of pregnancy and for offspring health. Maternal obesity has severe consequences in offspring cardiovascular health, insulin sensitivity, and development of organs. Mechanisms that facilitate this programming effect in offspring beginning from gestation until lactation are being unearthed as research progresses. Further, supplementation of certain diets and exercise interventions negated the effect of maternal obesity in offspring.

Recent evidences show that obesity is altered by microbiome and microbiome has been shown to be altered by the birth outcome (148). Thus, it will be interesting to investigate microbiome and maternal interventions that have effects on offspring. Though programming studies have been done to understand the role of nutritional interventions in tackling maternal obesity, their role in epigenetic mechanisms is not completely understood and needs further research. Also, epigenetic mechanisms behind exercise interventions are still in their infancy. For example, studies have demonstrated hypermethylation of PGC-1α, but more studies are required to understand the entirety of epigenetic alterations with physical activity. Lastly, physical activity during the second and third trimesters reduces maternal serum C-reactive protein (CRP), an inflammatory marker; however, whether physical activity also reduces inflammatory markers in the offspring needs further investigation (149, 150).

Long-term effects of maternal obesity can also propagate to 2–3 generations, which highlights the importance of studying F1, F2, and F3 generations when using animal models. Since it is relatively difficult to assess the generational effects of maternal obesity in human subjects, more animal studies are suggested. Nonetheless, human studies following children of obese mothers from early childhood to adulthood can provide a better understanding of how obesity develops. Finally, the interventions reported in literature are split into two categories based on the physical activity and dietary interventions. It is important to perform comprehensive studies by combining nutritional and physical activity interventions to assess the benefits of such combinatorial approaches on mitigating the adverse effects of maternal obesity in offspring.

## Author Contributions

AS and V-MB-S drafted the manuscript. MP, JG, and LR edited the manuscript. LR takes full responsibility for the manuscript content. All authors contributed to the article and approved the submitted version.

## Conflict of Interest

The authors declare that the research was conducted in the absence of any commercial or financial relationships that could be construed as a potential conflict of interest.
